# A Text-Book of Practical Therapeutics

**Published:** 1890-10

**Authors:** 


A Text-Book of Practical Therapeutics. With especial reference to the Application of Remedial Measures to Disease and their Employment upon a Rational Basis. By Hobart Amory Hare, B. Sc., M. D., Clinical Professor of Diseases of Children and Demonstrator of Therapeutics in the University of Pennsylvania; Laureate of the Royal Academy of Medicine in Belgium; of the Medical Society of London; Member of the American Association of Physicians; Secretary of the Convention for the Revision of the United States Pharmacopeia of 1890; Physician to St. Agnes Hospital and the Dispensary of the Children's Hospital, Philadelphia. Cloth and leather, 8vo, pp. vi.—632. Philadelphia: Lea Brothers & Co. 1890.

This important work comes at an opportune time. The colleges have just opened and new students are seeking to enter upon the study of medicine. For them, and for older students also, the question of text-books is most important. We have some excellent and learned works on materia medica and therapeutics, but as Dr. Hare well says, they consider the subject as if the student were a skilled physician or experimental pharmacologist. It is for this reason that this department is considered so very difficult. It requires close familiarity with the knowledge of drugs to treat disease successfully, and a book that arranges this knowledge with a view to the needs of students, becomes at once popular. We have but to refer to the famous work of Ringer in illustration. The rapidity with which its various editions have been exhausted has shown the need it fulfils. After a careful examination of Dr. Hare's work, we venture the opinion that when it becomes known it will rival the other in popularity. With great judgment he has placed before us a resumé, if we may so speak, of the action of drugs and other remedies, and of the treatment of disease. The student is not confused by the continual reference to authorities, and his confusion is not confounded by the detail of conflicting experiments. He is told what we know to-day in the broad field of therapeutics, and nowhere, that we recall, in this information given with more care and judgment. The author seeks in all cases where there is want of definiteness to give a fair and proper interpretation of the present views without any special pleading in favor of this or that conclusion. The work is divided into four parts, as follows:

Part I. General therapeutic considerations.

Part II. Drugs.

Part III. Remedial measures other than drugs. Foods for the sick.

Part IV. Diseases. Table of doses and remedies. Index of drugs and remedial measures. Index of diseases and their treatment.

The work throughout is arranged in alphabetical order. Of this departure from the ordinary arrangement the author says:

> This has been done because it is desired to afford the reader a ready reference book to which he may turn at short notice for desired information, for at present the state of pharmacology is so unsettled that a true classification is impossible. Thus morphine may be classed by one writer as a nervous sedative, by another, as a sleep-producer, by a third, as a bitter substance, and by a fourth, as a respiratory depressant. Bromide of potassium can, with equal propriety, be called a spinal sedative or a cerebral sedative, or caffeine be classed as a cerebral stimulant, a circulatory stimulant, or a diuretic.

The book also contains the preparations of the British Pharmacopeia.

We cannot but believe that Dr. Hare has rendered a great service by the preparation of his work, and that it will receive the hearty indorsement of all teachers and students.

The volume is a beautiful specimen of the printer's art, and in this direction leaves nothing to be desired. F. H. P.

